# Automatic Vasculature Identification in Coronary Angiograms by Adaptive Geometrical Tracking

**DOI:** 10.1155/2013/796342

**Published:** 2013-10-22

**Authors:** Ruoxiu Xiao, Jian Yang, Mahima Goyal, Yue Liu, Yongtian Wang

**Affiliations:** ^1^Key Laboratory of Photoelectronic Imaging Technology and System, Ministry of Education of China, School of Optics and Electronics, Beijing Institute of Technology, Beijing 10081, China; ^2^Amity School of Engineering & Technology, Amity University, Noida, Uttar Pradesh 201303, India

## Abstract

As the uneven distribution of contrast agents and the perspective projection principle of X-ray, the vasculatures in angiographic image are with low contrast and are generally superposed with other organic tissues; therefore, it is very difficult to identify the vasculature and quantitatively estimate the blood flow directly from angiographic images. In this paper, we propose a fully automatic algorithm named adaptive geometrical vessel tracking (AGVT) for coronary artery identification in X-ray angiograms. Initially, the ridge enhancement (RE) image is obtained utilizing multiscale Hessian information. Then, automatic initialization procedures including seed points detection, and initial directions determination are performed on the RE image. The extracted ridge points can be adjusted to the geometrical centerline points adaptively through diameter estimation. Bifurcations are identified by discriminating connecting relationship of the tracked ridge points. Finally, all the tracked centerlines are merged and smoothed by classifying the connecting components on the vascular structures. Synthetic angiographic images and clinical angiograms are used to evaluate the performance of the proposed algorithm. The proposed algorithm is compared with other two vascular tracking techniques in terms of the efficiency and accuracy, which demonstrate successful applications of the proposed segmentation and extraction scheme in vasculature identification.

## 1. Introduction 

World Health Organization's survey of “The top ten causes of death” acknowledged the fact that coronary artery diseases (CADs) are the leading cause of human deaths worldwide. CADs were responsible for 7.25 million deaths in 2008, which accounted for 12.8% of the total deaths worldwide, and this number of deaths has been increasing ever since [1]. The X-ray angiography is an effective technique for imaging of the coronary artery and is considered as the “golden standard” for clinical observation of coronary anatomy and identification of vascular stenosis [2]. Therefore, it is widely used in clinical diagnosis and monitoring of disease. The coronary artery obtained from the X-ray angiograms can provide useful parameters for quantitative assessment and diagnosis of cardiovascular disease. Furthermore, the extraction of coronary arteries from the sequence of angiographic images is an important basis for heart motion analysis [3–5] and 3D vascular reconstruction [6–9]. However, fully automatic, robust, and accurate extraction of coronary artery from angiograms is still a challenging task so far. The main difficulties for the accurate vascular structure extraction or identification in angiograms are as follows: (1) the irregular gray level distribution of blood vessel due to the uneven perfusion of the contrast agent; (2) the nonvascular structures background interference including bones, catheters, and soft tissues; (3) the diversity of direction and width of the vessels; and (4) the commonly existing motion artifacts due to the heart motion and the presence of some pathological lesions.

In the past two decades, a few methods have been studied for the extraction of the blood vessels in angiographic images, such as morphology based methods [10], tracking based methods [11–16], multiscale based, methods [17, 18], edge detection methods, and registration based methods [19, 20]. Among them, the tracking-based methods proved to be very effective, which can detect coronary artery according to the local response of the angiograms and do not need to scan the whole image. Also, the methods extract the parameters including centerlines, diameter, or bifurcations using an adjustable pattern element to fit incremental section tracking procedures. 

For conventional tracking-based algorithm, adapted diameter measurement [11] often suffers from artifacts due to nonuniform contrast distribution of the contrast materials. Diameter measurement approaches have been applied to the single segment and the full vascular network and can produce acceptable quality in the coronary artery extraction [21]. And also, certain new template-based techniques have been introduced [14–16] to fit the coronary artery structure in the tracking procedure, such as rectangular or circular templates [15], Gabor filters [14], and Gaussian kernels [16]. The template-based model construction is known to be complicated and time consuming. Most of them need to create massive templates according to the variation of diameter and direction of coronary artery.

 To reduce the calculation complexity, an alternative solution of intensity ridge detection is utilized to approximate the medial axes of the coronary artery. Aylward and Bullitt [12] proposed a centerline tracking algorithm based on ridge detection in multi-scale space, which is constructed by extracting the ridge using eigen decomposition of the Hessian matrix. Zamani Boroujeni et al. [13] employed a ridge scanning scheme for reliable identification of the vessel points and calculating the magnitude by adaptive look-ahead distance method. However, due to the effects of low image quality and noise, ridgeline cannot be exactly located at the geometrical center of coronary artery.

In this paper, an adaptive geometrical vessel tracking (AGVT) algorithm is proposed for the automatic extraction of coronary artery from angiograms. There are two main contributions of this study. First, an automatic initialization algorithm including location and direction of the seed point is proposed. Second, ridge points are recursively detected in consecutive scanlines, which are then adjusted to the geometrical centers according to the estimated diameters. The proposed method does not require any human intervention beforehand and can simultaneously estimate the parameters for quantitative analysis of vasculature including centerlines, diameter, and bifurcations.

## 2. Methods

The proposed AGVT algorithm is composed of three main steps: ridge enhancement, seeds determination, and adaptive tracking. The brief description of the calculation procedures is as follows.

1. Ridge enhancement: the angiogram is first convoluted with a Gaussian kernel with different standard deviation. For each scale of convolution, the blood vessel is enhanced by combination of eigenvalue response of the Hessian matrix, and the maximum responses for each level of scale space are extracted as the RE image. The enhancement procedure guarantees that the background is suppressed and the vascular structures are highlighted in the RE image.
2. Seeds determination: the seed points are detected by scanning of local maximum, for which the intensity is brighter than other points in the local area. The forward and backward tracking directions of the seeds are designed to detect the neighboring ridge points.
3. Adaptive tracking: rough centerlines are extracted through sequentially detecting ridge point in RE image, which are then further refined adaptively to the geometric center according to the distance between the vascular boundaries and current calculating point. If the bifurcation is identified by discriminating connecting relationship of the tracked ridge points, the tracking will be divided into two paths for each branch. Termination criteria are assigned to ensure that the tracking is within the region of coronary artery without repetition. Finally, all the detected centerlines are merged and smoothed, and also false tracking results are removed.

### 2.1. Ridge Enhancement

In this paper, we use a multiscale vascular enhancement filter (MVEF) [22] to obtain the RE image. It enhances all the tubular targets along the centerline and fades out the background. Suppose the *H*(*p*) is the Hessian matrix of a point *p* on image *I*; then, the Hessian matrix of the each pixel can be calculated as follows:

(1) $$\begin{array}{c} H\left(p\right)=\left(\begin{array}{cc} I_{xx}\left(p\right) & I_{xy}\left(p\right)\\ I_{yx}\left(p\right) & I_{yy}\left(p\right) \end{array}\right), \end{array}$$

where subscripts *x* and *y* denote the second order derivate along *x* or *y* direction. The eigenvalues of the matrix are denoted by *λ*_1_ and *λ*_2_ (*λ*_1_ ≤ *λ*_2_). According to the analysis of [22], different eigenvalues of the Hessian matrix are corresponding to different types of structure, such as plate-like, blob-like, tubular, and noises.

In order to detect different size of vessels, Gaussian kernel with different standard deviation is usually convolved with the angiogram [23, 24]. For a certain scale *σ*, the intensity differential of the point *p* is expressed as follows:

(2) $$\begin{array}{c} \frac{\partial }{\partial x}I\left(p,\sigma \right)=\sigma^{\gamma }I\left(p\right)*\frac{\partial }{\partial x}G\left(0,\sigma \right), \end{array}$$

where *γ* is the normalization coefficient as defined in [24] and *G*(0, *σ*) denotes a Gaussian function with the mean of 0 and standard deviation of *σ*. The *σ* of the Gaussian kernel is designed as a variant value to enhance different scale of vessels. In implementation, *σ* is usually designed as a value between the maximum and minimum size of the vascular diameter to be enhanced. The enhancement response of each pixel in scale space is computed, and the maximum response is then utilized as the final enhancement result. The enhancement response *V*(*p*) of pixel *p* can be calculated as follows:

(3) V(p,σ)={0,if  λ2<0exp⁡(−λ122α2λ22)[1−exp⁡(−λ12+λ222β2)]otherwise,V(p)=max⁡σmin⁡≤σ≤σmax⁡V(p,σ),

where *α* and *β* are control parameters, while [*σ*_min⁡_, *σ*_max⁡_] is the size of the scale space. 

### 2.2. Seeds Determination

#### 2.2.1. Seed Points

After the ridge enhancement, the local maximum points in the gray level space of the RE image are located on the ridgelines, and they are detected as the initial positions for the tracking. According to [25], if a point (*x*, *y*) is a local maximum, then its gradient is equal to zero and its Hessian matrix is negative. However, the points satisfied with these two conditions are usually with float coordinates. Therefore, the seed points need to be interpolated according to their neighboring coordinates.

For any pixel (*x*, *y*) and its neighbor pixels (*x* + 1, *y*), (*x*, *y* + 1), (*x* + 1, *y* + 1), if there is a point (*ξ*, *η*)  (*x* < *ξ* < *x* + 1,  *y* < *η* < *y* + 1) that meets the conditions that ∇(*ξ*, *η*) = 0 and *λ*_1_(*ξ*, *η*) < 0, *λ*_2_(*ξ*, *η*) < 0, then (*ξ*, *η*) is a local maximum on the image. According to the bilinear interpolation equation

(4) f(ξ,η)=[x+1−ξx−ξ] ×[f(x,y)f(x,y+1)f(x+1,y)f(x+1,y+1)] ×[y+1−ηy−η],

we have

(5) ∇(ξ,η)=[x+1−ξx−ξ] ×[∇(x,y)∇(x,y+1)∇(x+1,y)∇(x+1,y+1)] ×[y+1−ηy−η]=0,λi(ξ,η)=[x+1−ξx−ξ] ×[λi(x,y)λi(x,y+1)λi(x+1,y)λi(x+1,y+1)] ×[y+1−ηy−η]<0, (i=1,2).

The solutions of (5) are implicit; hence, the approximate solutions can be solved by calculating the continuity of the image and its differential information. If a point satisfied the following equations:

(6) ∇(x,y)∇(x+1,y+1)<0  or∇(x+1,y)∇(x,y+1)<0,λi(x+m,y+n)<0, (i=1,2, m=0,1, n=0,1)

there will be a point (*ξ*, *η*)  (*x* < *ξ* < *x* + 1,  *y* < *η* < *y* + 1), which satisfies the conditions ∇(*ξ*, *η*) = 0 and *λ*_1_(*ξ*, *η*) < 0, *λ*_2_(*ξ*, *η*) < 0. Due to the continuity of image, (*x*, *y*) is an approximate solution of (*x*, *y*).

Since there are still numerous noises with low gray value in RE image, the extracted seed points are refined by an intensity threshold *τ*. If the intensity value of a candidate seed point is below the predefined threshold, it will be discarded.

#### 2.2.2. Tracking Directions

In this section, the initial tracking directions of the detected seed points are obtained in RE image. Suppose that *p* is a detected seed point, while *p* ^+^ and *p* ^−^ are its neighboring forward (with greater abscissa value) and backward (with smaller abscissa value) points. Then the forward tracking direction **u**(*p* ^+^) and backward tracking direction **u**(*p* ^−^) of *p* can be formulated as

(7) $$\begin{array}{c} u\left(p^{+}\right)=\frac{p^{+}-p}{||p^{+}-p||},\\ u\left(p^{-}\right)=\frac{p^{-}-p}{||p^{-}-p||}. \end{array}$$

**Figure 1:**
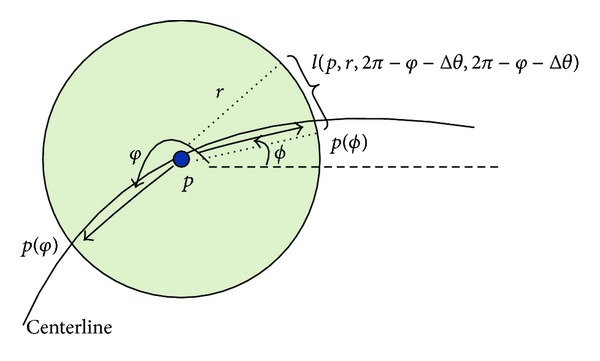
Determination of the forward and backward tracking directions for a seed point.

As shown in Figure 1, for a seed point *p* with coordinate (*x*_*p*_, *y*_*p*_), we define a circle with radius *r* centered at *p*, and the points *p*(*θ*)  (*p*(*θ*) = *p*(*x*_*θ*_, *y*_*θ*_)) on the circle can be expressed as parametric equations as follows:

(8) $$\begin{array}{c} x_{\theta }=x_{p}+r\cos\left(\theta \right),\;y_{\theta }=y_{p}+r\sin\left(\theta \right),\;0\leq \theta <2\pi . \end{array}$$

As there are two intersections between the circle and the centerline, the intersection points *p*(*φ*) and *p*(*ϕ*) hence can be detected by calculating gray level difference along the path of the circle. Here, the point *p*(*φ*) can be detected by finding the maximum on the circle as follows:

(9) $$\begin{array}{c} \varphi =\underset{\theta \in \left[0,2\pi \right)}{\arg\max}\;I\left(p\left(\theta \right)\right). \end{array}$$

Obviously, *p*(*φ*) is one of the points *p* ^+^ or *p* ^−^, while *p*(*ϕ*) can be detected by finding the local maximum on the local arc defined by *φ*:

(10) $$\begin{array}{c} \phi =\underset{\theta \in [2\pi -\varphi -\Delta \theta ,2\pi -\varphi +\Delta \theta ]}{\arg\max}I\left(p\left(\theta \right)\right), \end{array}$$

where *φ* and *ϕ* represent the forward and backward tracking angle of pixel *p*. [2*π* − *φ* − Δ*θ*, 2*π* − *φ* − Δ*θ*] represents the searching interval. The condition of cos⁡(*φ*) ≥ 0 means that *p*(*φ*) is located at the right side of *p*.

Since noise is widely scattered over RE image, it is necessary to refine the seed points from the noise. Here, a rectangle with size of *Lx* × *Ly* is constructed on the center of each seed point. Then, if the average intensity within the defined rectangle is below a predefined threshold *τ*, this seed point is removed.

#### 2.2.3. Adaptive Tracking

*(a) Centerline Updating and Diameter Measurement.* Due to the nonuniform distribution of injected contrast, the ridgeline of vessel cannot be accurately located at the geometrical center of the vessel in angiographic image. Hence, in this paper, we first extract the ridge point in RE image and then adjust it to the geometrical center by geometric symmetry property of the boundaries of the vascular structures.

**Figure 2:**
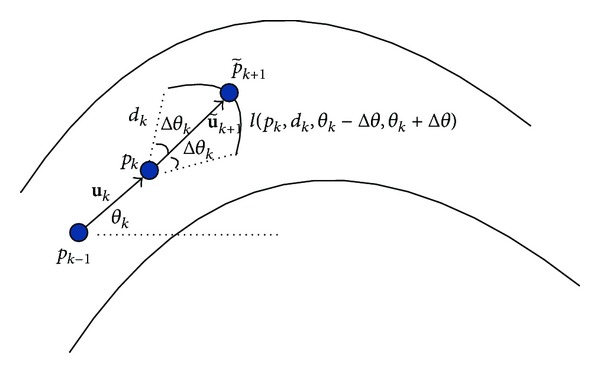
The process of ridge tracking.

Figure 2 illustrates the process of ridge tracking. The location and tracking angle of current centerline point are denoted by *p*_*k*_ and *θ*_*k*_; then, the relationship between the tracking direction **u**_*k*_ and tracking angle *θ*_*k*_ is defined as follows:

(11) $$\begin{array}{c} u_{k}=\left(\cos\theta_{k},\sin\theta_{k}\right). \end{array}$$

To detect the ridge point ${\tilde{p}}_{k+1}$, the intensities of the pixels located on arc *l*(*p*_*k*_, *d*_*k*_, *θ*_*k*_ − Δ*θ*, *θ*_*k*_ + Δ*θ*) are enumerated and compared. And the local maximum with intensity equal to the ridge point ${\tilde{p}}_{k+1}$ can be found as follows:

(12) $$\begin{array}{c} I\left({\tilde{p}}_{k+1}\right)=\underset{p\in l\left(d_{k},\theta_{k}-\Delta \theta ,\theta_{k}+\Delta \theta \right)}{\max}I\left(p\right), \end{array}$$

where 2Δ*θ*_*k*_ is the search scope and *d*_*k*_ is the calculating step size.

Then, the initial tracking direction ${\tilde{u}}_{k+1}$ can be defined by *p*_*k*_ and ${\tilde{p}}_{k+1}$ as follows:

(13) $$\begin{array}{c} {\tilde{u}}_{k+1}=\frac{{\tilde{p}}_{k+1}-p_{k}}{||{\tilde{p}}_{k+1}-p_{k}||}. \end{array}$$

**Figure 3:**
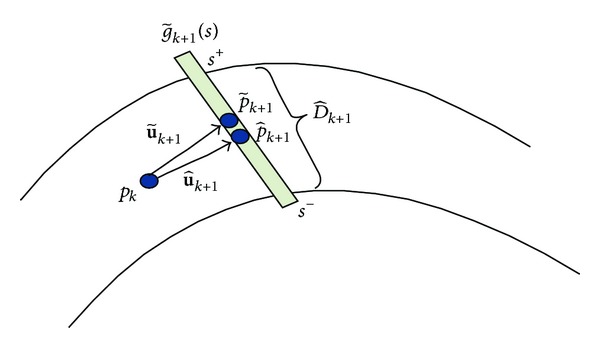
Centerline point adjustment and diameter measurement.

Let ${\tilde{p}}_{k+1}$ and ${\hat{p}}_{k+1}$ represent the detected ridge point and its corresponding geometrical centerline point. As shown in Figure 3, the intensity profile ${\tilde{g}}_{k+1}\left(s\right)$ is defined by the scanline of ${\tilde{p}}_{k+1}$, which is perpendicular to ${\tilde{u}}_{k+1}$. And *s* is denoted by the curve parameter of ${\tilde{g}}_{k+1}\left(s\right)$. 

 Hence, two estimated edge points ${\tilde{g}}_{k+1}\left(s^{\;+}\right)$ and ${\tilde{g}}_{k+1}\left(s^{\;-}\right)$ can be detected according to the gradient information of ${\tilde{g}}_{k+1}\left(s\right)$:

(14) $$\begin{array}{c} \nabla \left|{\tilde{g}}_{k+1}\left({\tilde{s}}^{\;+}\right)\right|=\underset{0<s<D}{\max}\nabla \left|{\tilde{g}}_{k+1}\left(s\right)\right|,\\ \nabla \left|{\tilde{g}}_{k+1}\left({\tilde{s}}^{\;-}\right)\right|=\underset{-D<s<0}{\max}\nabla \left|{\tilde{g}}_{k+1}\left(s\right)\right|, \end{array}$$

where the parameter *D* is the defined as the search size, while + and − denote the upward and downward direction of ${\tilde{p}}_{k+1}$, respectively.

 Then, we count the average gray value of the vessel points and background to obtain the two corresponding edge points ${\tilde{g}}_{k+1}\left({\tilde{s}}^{\;+}\right)$ and ${\tilde{g}}_{k+1}\left({\tilde{s}}^{\;-}\right)$. The average gray value ${\tilde{V}}_{k+1}$ of the vessel points in the profile ${\tilde{g}}_{k+1}\left(s\right)$ is calculated as follows:

(15) $$\begin{array}{c} {\tilde{V}}_{k+1}=\frac{1}{{\tilde{s}}^{\;+}-{\tilde{s}}^{\;-}}\underset{{\tilde{s}}^{\;-}<s<{\tilde{s}}^{\;+}}{\sum }{\tilde{g}}_{k+1}\left(s\right). \end{array}$$

And the average gray value of background ${\tilde{B}}_{k+1}$ in ${\tilde{g}}_{k+1}\left(s\right)$ is calculated as follows:

(16) $$\begin{array}{c} {\tilde{B}}_{k+1}=\frac{1}{2D-\left({\tilde{s}}^{\;+}-{\tilde{s}}^{\;-}\right)}\left(\underset{-D<s<{\tilde{s}}^{\;-}}{\sum }{\tilde{g}}_{k+1}\left(s\right)+\underset{{\tilde{s}}^{\;+}<s<D}{\sum }{\tilde{g}}_{k+1}\left(s\right)\right). \end{array}$$

The two edge points ${\tilde{g}}_{k+1}\left({\hat{s}}^{\;+}\right)$ and ${\tilde{g}}_{k+1}\left({\hat{s}}^{\;-}\right)$ with gray value less than $\left(1/2\right)\left({\tilde{V}}_{k+1}+{\tilde{B}}_{k+1}\right)$ on either side of ${\tilde{p}}_{k+1}$ are identified. Then, the geometrical centerline point ${\hat{p}}_{k+1}$ corresponding to ${\tilde{p}}_{k+1}$ can be calculated as follows:

(17) $$\begin{array}{c} {\hat{p}}_{k+1}={\tilde{g}}_{k+1}\left(\frac{{\hat{s}}^{\;+}+{\hat{s}}^{\;-}}{2}\right). \end{array}$$

According to (13), the adjusted tracking direction ${\hat{u}}_{k+1}$ of ${\hat{p}}_{k+1}$ can be then updated as

(18) $$\begin{array}{c} {\hat{u}}_{k+1}=\frac{{\hat{p}}_{k+1}-p_{k}}{||{\hat{p}}_{k+1}-p_{k}||}. \end{array}$$

Once the positions of the two edge points ${\tilde{g}}_{k+1}\left({\hat{s}}^{\;+}\right)$ and ${\tilde{g}}_{k+1}\left({\hat{s}}^{\;-}\right)$ are determined, the diameter ${\hat{D}}_{k+1}$ of the centerline point ${\hat{p}}_{k+1}$ can be measured as

(19) $$\begin{array}{c} {\hat{D}}_{k+1}=\left|{\hat{s}}^{\;+}-{\hat{s}}^{\;-}\right|. \end{array}$$

**Figure 4:**
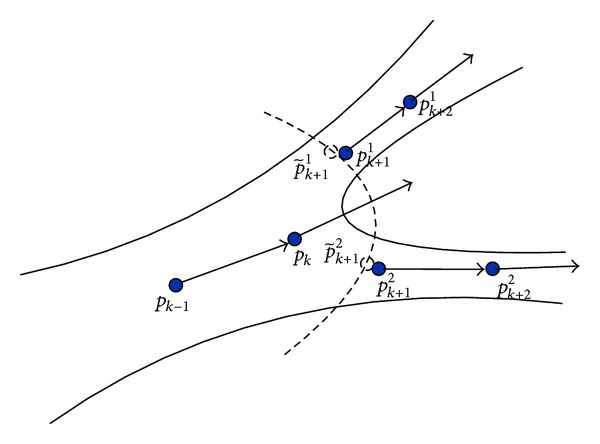
Bifurcation identification.

*(b) Bifurcations Identification.* As shown in Figure 4, *p*_*k*−1_ and *p*_*k*_ are two detected centerline points in the previous tracking process. If *p*_*k*_ is a bifurcation, then two ridge points ${\tilde{p}}_{{\;}_{k+1}}^{1}$and ${\tilde{p}}_{{\;}_{k+1}}^{2}$ can be found on RE image at each branch of the vessel due to the process of   MVEF. According to the adaptive tracking described in the previous section, we are able to get two geometrical centerline points *p*_*k*+1_^1^ and *p*_*k*+1_^2^. Thereafter, the tracking process is proceeded in the two directions of **u**_*k*+1_^1^ = (*p*_*k*+1_^2^ − *p*_*k*_)/||*p*_*k*+1_^2^ − *p*_*k*_|| and **u**_*k*+1_^2^ = (*p*_*k*+1_^2^ − *p*_*k*_)/||*p*_*k*+1_^2^ − *p*_*k*_||. 

*(c) Termination Criteria.* The tracking procedure starts from a randomly selected seed point and then iteratively extends to the other seed points. Three termination rules are defined to prevent the tracking path from going out of the image range, or being repeatedly calculated.

1. If the detected centerline point goes beyond the scope of the image.
2. If the detected centerline point goes beyond the scope of the vessel.
3. If the detected centerline point intersects an extracted centerline.

Condition (1) is designed to guarantee that both abscissa value and ordinate value of current calculating point are within the border of the image while condition (2) is designed to ensure that the gray value of current calculating pixel is within the range of the vascular boundaries. Condition (3) is devised to determine if current calculating pixel is being detected or not.

A seed point tracking process is stopped immediately if any of the above three conditions occurs, and a new seed point tracking procedure will be launched. By setting these three conditions, it can be guaranteed that the tracking procedure will be carried out in the region of the blood vessels without repetition.

*(d) Vasculature Refinement.* After all discrete incremental sections are obtained from the angiographic image, vasculature refinement needs to be done to remove the false tracking noise, such as hair-like noise and discrete small edges. All the discrete centerlines are merged into several connected centerline sets, and the short false tracking results are removed by a predefined length threshold. Then, the vessel structure with the largest connected components is reserved as the tracking result. To avoid “jagged” tracking phenomenon, the cardinal spline interpolation [26] is used to preserve the smoothness of the tracked paths.

## 3. Results and Discussion

To validate the performance of the proposed vascular extraction method, series of experiments are designed and tested on both synthetic data and real clinical angiograms. And the comparative evaluation of the results demonstrates the efficiency of the proposed method over the several existing methods. Our tracking algorithm is implemented in Microsoft Visual Studio 2010 on an Intel Core i7 PC (with CPU 3.5 G and 16 G memory), and all the simulated angiographic images are with the resolution of 512 × 512.

### 3.1. Synthetic Data

In order to quantitatively evaluate the performance of the proposed method, a series of angiograms with defined vascular structures are designed and simulated. The angiograms are simulated by projecting 3D synthetic cylindrical segments onto the image plane according to the perspective projection model [27] of the angiography system as developed in [13, 28, 29]. In order to simplify the simulation procedure, the background image is acquired prior to the injection of contrasting substance, so there is no visible vessel or catheter in the obtained image. 

The projection intensity of vessel segment model adopted for the simulation comes from [27], which can be defined as follows:

(20) $$\begin{array}{c} p\left(x\right)=2\mu \sqrt{r^{2}-x^{2}},\;\left|x\right|\leq r, \end{array}$$

where *x* is the distance between current point and tentative centerline point, *r* is the radius of vessel, *μ* is the linear attenuation coefficient, and *p*(*x*) is the intensity of the projected point. 

**Figure 5:**
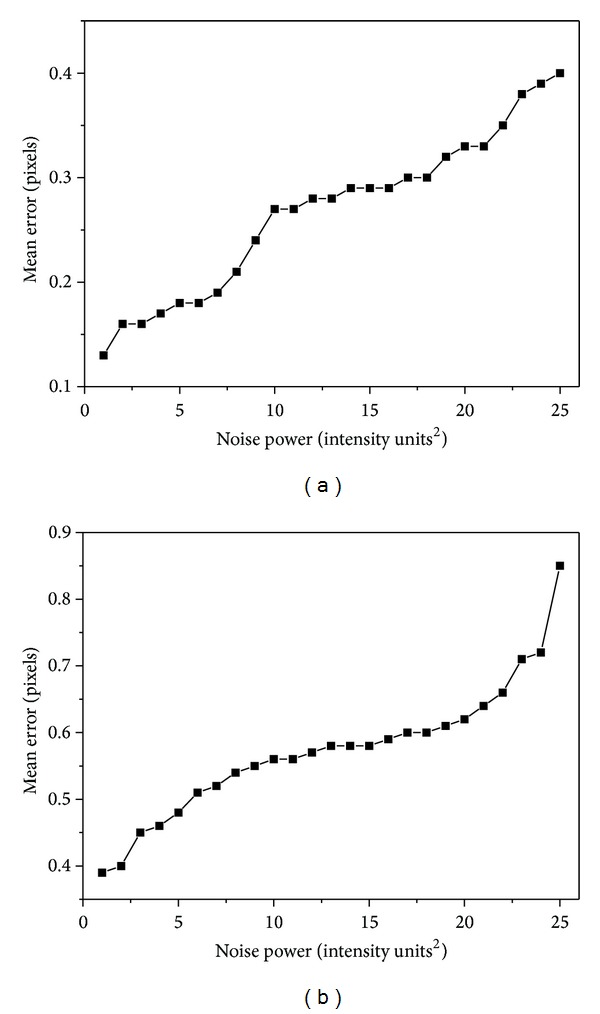
Error distribution. (a) Centerline error. (b) Diameter error.

To test the proposed algorithm over noise interference, 25 angiograms containing a single vessel segment with different additive Gaussian noise are generated. The proposed algorithm then proceeds on the simulated image, and the extracted centerline and diameter are compared with the predefined vessel, and the error distributions over different noise scale for the centerline and diameter estimation are shown in Figures 5(a) and 5(b), respectively. From Figure 5 it can be seen that with the adding of noise power, the mean error of the centerline and diameter estimations increase gradually. Specifically, the mean error for centerline estimation ranges from 0.13 pixels to 0.33 pixels for noise power between 1 and 20; while for the diameter, the estimated errors range from 0.39 pixels to 0.62 pixels for noise power between 1 and 20. For noise power of 25, the image is considerably corrupted; however, the tracking centerline and diameter estimation errors are less than 0.4 and 0.9 pixels. It is obvious that the proposed method is every effective and robust even under terrible image qualities.

In order to simulate the vascular stenosis, the diameter is adjusted by a designed exponentially decaying function as formulated as follows [29]:

(21) $$\begin{array}{c} D\left(l\right)=D_{0}\exp\left(-al\right)-\frac{c}{\sigma }\exp\left[-\left(\frac{\pi {\left(l-\mu \right)}^{2}}{{\left(\sigma c\right)}^{2}}\right)\right],\\ c=D_{0}\exp\left(-a\mu \right), \end{array}$$

where *D*_0_ is an initial diameter and *a* is taper coefficient. *μ* and *σ* are Gaussian function parameters. For this calculation, at the stenosis part (*l* = *μ*), 100% stenosis can be simulated by setting *σ* = 1. Stenosis rate of vessel can computed as follows:

(22) $$\begin{array}{c} \text{stenosis}=\frac{1}{\sigma }. \end{array}$$

**Figure 6:**
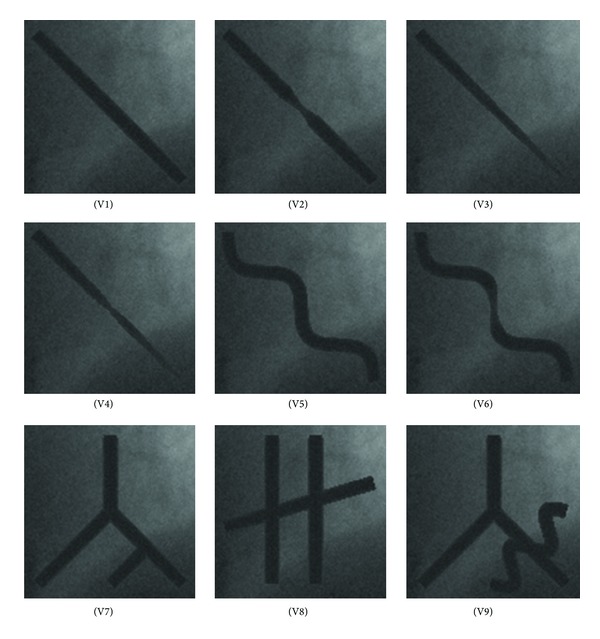
Nine types of synthetic arterial segments.

According to (21) and (22), nine types of arterial segments with stenosis are generated, as shown in Figure 6, and the performance of the proposed algorithm is compared with the other two existing centerline tracking algorithms. To simplify the following description, nine types of arterial segments named V1 to V9 are designed as follows.

(1) V1: vessel with rough parallel boundaries; (2) V2: vessel with the diameter decreased gradually; (3) V3: vessel with curved boundaries, for which the centerline of the vessel is computed by connecting two periods sine functions; (4) V4: V1 with 50% stenosis; (5) V5: V2 with 50% stenosis; (6) V6: V3 with 50% stenosis; (7) V7: vessels with bifurcations; (8) V8: vessels with overlaps; (9) V9: vessels with bifurcation and overlap.

The tracking results of the three algorithms are listed in Table 1, which contains the maximum, minimum, mean, and RMS (root mean square) of the estimated centerline error. The mean errors are 0.34, 1.27, and 0.48 pixels for the proposed algorithm, Aylward, and Boroujeni algorithms, respectively. The proposed method represents 73.22% and 29.17% improvement over Aylward and Boroujeni algorithms. For the most complex structure V9, the mean tracking error for the proposed algorithm is 0.41 pixels, while it is 1.61 pixels and 0.87 pixels for Aylward and Boroujeni algorithms. The proposed method represents 74.53% and 39.87% improvement over Aylward algorithm and Boroujeni algorithm. Obviously, the proposed algorithm provides a more accurate scheme for coronary artery extraction and identification in X-ray angiograms. 

The above experiments are repeatedly tested on 45 groups of synthetic images, for which each type of the vessel structure is randomly generated for five times. For these experiments, the average computational times are 3.66 s, 3.82 s, and 3.85 s for Aylward, Boroujeni, and the proposed algorithms, respectively. It shows that all these three algorithms take roughly the same computational time for the vessel extraction in angiograms. The computation efficiency can be further improved by integrating GPU based implementations in the future.

### 3.2. Clinical Angiograms

**Figure 7:**
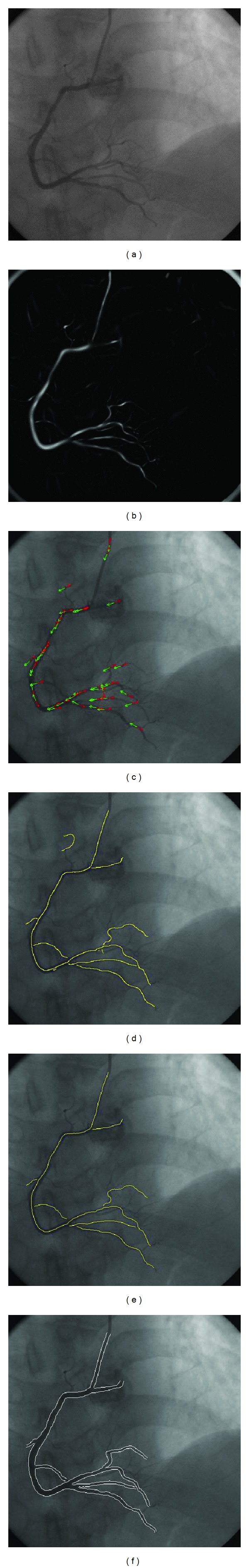
Experimental results of the proposed algorithm on a routine coronary angiogram. (a) The original image. (b) MVEF enhancement. (c) Seed determination. (d) Tracking results of (c). (e) Vasculature refinement of (d). (f) Boundaries of coronary artery.

**Figure 8:**
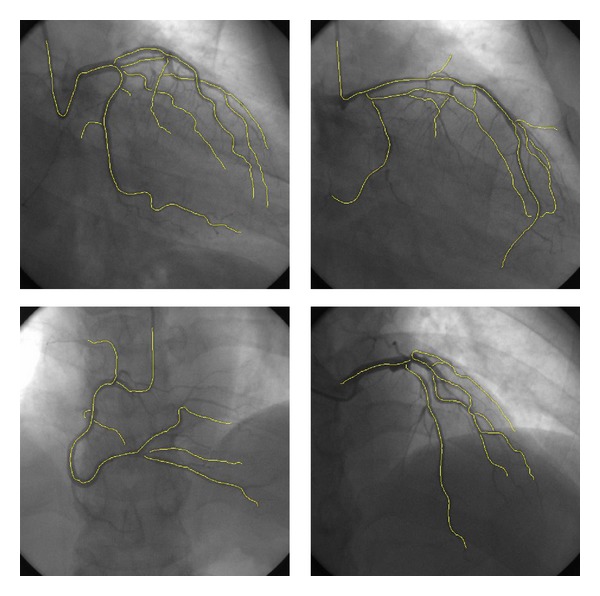
The other four examples of the tracked results for the proposed method.

In order to investigate the applicability and efficiency of the proposed algorithm, the proposed method is also applied to 20 coronary angiograms which were randomly selected from a database routinely acquired from the Philips Digital Imaging System at the Beijing Chaoyang Red-Cross Hospital during invasive catheterization procedures. Here, the experimental result of a random angiogram (Figure 7(a)) is selected to illustrate each step of the calculation.  Figure 7(b) is the RE image obtained through the ridge enhancement operation of Figure 7(a), while Figure 7(c) shows the extracted seed points and initial tracking directions, where the red arrows represent the forward directions, while the green arrows depict the backward directions.  Figure 7(d) shows the tracking results of Figure 7(c), and Figure 7(e) gives the vasculature refinement results of Figure 7(d).  Figure 7(f) shows the boundaries of the extracted vessels. It can be seen from the images that through the process of MVEF, vessel is highlighted and the background is suppressed in RE image, and it serves as useful auxiliary information for the followed tracking steps. Through the seed determination of the proposed algorithm, it provides a set of seed points on the vascular centerline, and their directions are roughly parallel to the path of the vessels. From the figures, it can be seen that the proposed AGVT algorithm can extract main structure of coronary artery.  Figure 8 shows four representative tracking results from these 20 angiograms, and we can see that the main structure of the coronary arteries can also be extracted by the proposed algorithm.

**Figure 9:**
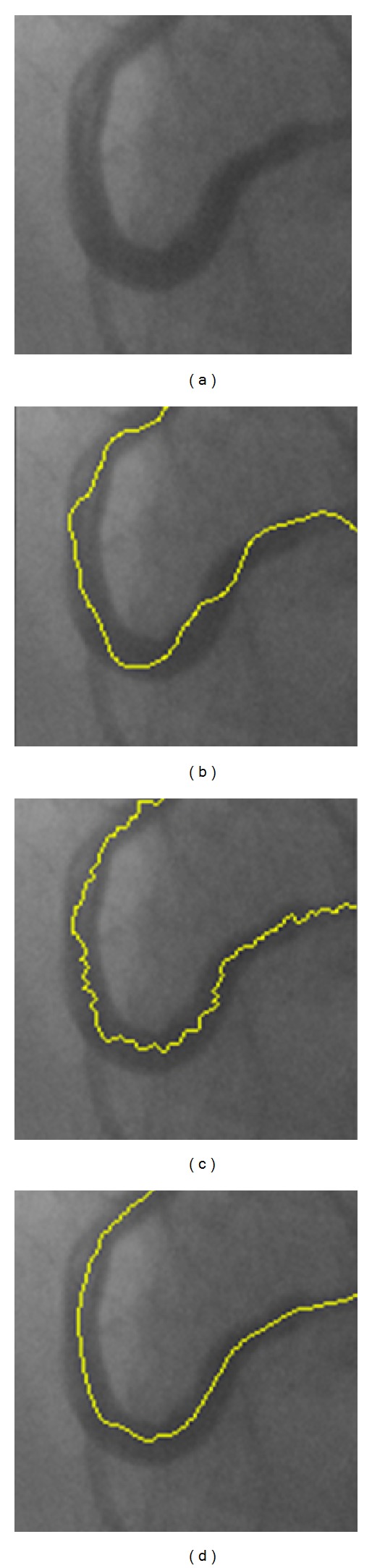
Partial enlargement of the vasculature extraction results. (a) The original image. (b) Results of Aylward algorithm. (c) Results of Boroujeni algorithm. (d) Results of the proposed algorithm.

Figure 9(a) shows a section of curved vessel for which the gray distribution is irregular. Figures 9(b), 9(c), and 9(d) show the tracking results of Aylward, Boroujeni, and the proposed algorithms, respectively. It can be seen that the tracking result of Aylward algorithm is more likely to change with the variation of gray level, which hence is more dependent on the gray level distribution of the angiogram. For Boroujeni algorithm, the detected centerline jitters significantly because it tracks the ridgeline rather than the geometrical center of the vascular structures. The proposed algorithm works better than the other algorithms as it adaptively extracts the geometrical centerline of the coronary artery and is more robust in processing irregular vascular segments.

**Figure 10:**
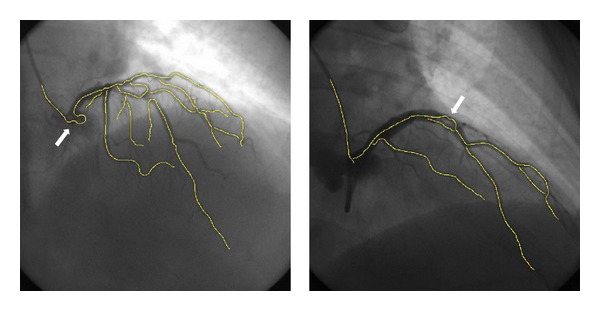
Two examples of challenging angiograms on which the proposed algorithms partially failed. The white arrows point to the partial failure tracking areas.

Despite the advances of the proposed algorithm demonstrated in this paper, Figure 10 shows two challenging angiograms from the set of 20 angiograms on which the proposed algorithm is only partially successful. It can be seen from Figure 10 that the proposed algorithm is failed for areas with overflowing (a) or incomplete filling (b) of the contrast agents in the angiograms. Up to know, segmentation of the vessels in such an angiogram is still a very challenge task.

## 4. Conclusions

The major contribution of this proposed algorithm is providing an automatic AGVT framework for coronary artery extraction by putting forward three major key steps named ridge enhancement, seed determination, and adaptive tracking. The proposed algorithm makes three key advances. First, considerable validated seed points and their tracking directions are automatically detected from angiograms, and it ensures the tracking affected without the interference of human factors. Second, it tracks coronary artery from angiograms without constructing templates, and, hence, the calculation complexity is greatly reduced. Third, the extracted ridge points are adaptively adjusted to the corresponding geometrical center, and it has been shown to work well with angiograms of low image quality. From the experiments on synthetic data and real angiograms, it can be stated that the proposed AGVT algorithm is more accurate and stable than the current widely used algorithms.

## Figures and Tables

**Table 1:** Quantitative comparison of Aylward, Boroujeni and the proposed algorithms.

Vessels	Aylward algorithm				Boroujeni algorithm				Proposed algorithm			
	Max	Min	Mean	RMS	Max	Min	Mean	RMS	Max	Min	Mean	RMS
V1	1.68	0.04	0.48	0.62	2.28	0.00	0.40	0.60	0.59	0.02	0.27	0.30
V2	2.71	0.02	1.06	1.30	2.82	0.00	0.39	0.65	0.67	0.01	0.28	0.32
V3	3.66	0.06	0.84	1.10	1.19	0.00	0.32	0.44	1.09	0.01	0.28	0.37
V4	3.69	0.03	1.24	1.50	2.21	0.02	0.40	0.64	0.55	0.01	0.21	0.25
V5	2.60	0.26	1.64	1.80	0.96	0.02	0.48	0.55	0.91	0.01	0.41	0.48
V6	2.52	0.24	1.62	1.70	2.10	0.00	0.63	0.80	1.31	0.01	0.41	0.53
V7	3.43	0.04	1.60	1.75	1.94	0.04	0.34	0.49	1.33	0.06	0.29	0.38
V8	3.27	0.02	1.38	1.57	2.07	0.02	0.52	0.86	1.64	0.09	0.47	0.62
V9	3.99	0.22	1.61	1.97	2.67	0.01	0.87	0.87	1.31	0.01	0.41	0.53

Mean	3.06	0.10	1.27	1.48	2.03	0.01	0.48	0.66	1.04	0.03	0.34	0.42
